# Association of *APOE* gene polymorphism with lipid profile and coronary artery disease in Afro-Caribbeans

**DOI:** 10.1371/journal.pone.0181620

**Published:** 2017-07-20

**Authors:** Laurent Larifla, Christophe Armand, Jacqueline Bangou, Anne Blanchet-Deverly, Patrick Numeric, Christiane Fonteau, Carl-Thony Michel, Séverine Ferdinand, Véronique Bourrhis, Fritz-Line Vélayoudom-Céphise

**Affiliations:** 1 Research Group Clinical Epidemiology and Medicine, ECM/L.A.M.I.A EA 4540, University of Antilles, Pointe-à-Pitre, France; 2 Department of Cardiology, University Hospital of Guadeloupe, Pointe-à-Pitre, France; 3 Department of Medical Information and Public Health, University Hospital of Guadeloupe, Pointe-à-Pitre, France; 4 Biochemistry Unit, University Hospital of Guadeloupe, Pointe-à-Pitre, France; 5 Department of Internal Medicine Unit, University Hospital of Martinique, Fort-de France, France; 6 Biochemistry Unit, University Hospital of Martinique, Fort-de France, France; 7 Department of Medicine, University Hospital of Guadeloupe, Pointe-à-Pitre, France; 8 Department of Endocrinology and Diabetology, University Hospital of Guadeloupe, Pointe-à-Pitre, France; Duke University, UNITED STATES

## Abstract

**Objectives:**

Apolipoprotein E gene (*APOE*) polymorphism is associated with the lipid profile and cardio-vascular disease. However, these relationships vary between ethnic groups.

We evaluated, for the first time in an Afro-Caribbean population, the distribution of *APOE* polymorphisms and their associations with coronary artery disease (CAD), the lipid profile and other cardio-metabolic risk factors.

**Methods:**

We studied 712 Afro-Caribbean subjects including 220 with documented CAD and 492 healthy subjects. TaqMan assays were performed to genotype rs7412 and rs429358, the two variants that determine the *APOE* alleles ε2, ε3 and ε4. The association between *APOE* genotype and the lipid profile was analysed by comparing ε2 carriers, ε3 homozygotes and ε4 carriers.

**Results:**

The frequencies of ε2, ε3 and ε4 in the overall sample were 8%, 70% and 22%, respectively. CAD was not associated with *APOE* polymorphism. The total cholesterol level was higher in ε4 carriers compared with ε2 carriers: 5.07 vs 4.59 mmol/L (P = 0.016). The LDL-cholesterol level was lower in *APOE* ε2 carriers compared with ε3 homozygotes and ε4 carriers: 2.65 vs 3.03 and 3.17 mmol/L, respectively (p = 0.002). The total cholesterol/HDL-cholesterol and LDL-cholesterol/HDL-cholesterol ratios were similar in the three allelic groups. *APOE* polymorphism was not associated with diabetes, hypertension, waist circumference or body mass index.

**Conclusions:**

Our results indicate that *APOE* gene polymorphism is associated with the lipid profile but not with CAD in Afro-Caribbean people. This lack of association with CAD may be explained by the low atherogenic profile observed in ε4 carriers, which may warrant further investigation.

## Introduction

Apolipoprotein E (ApoE) is a polymorphic glycoprotein that plays a multifunctional role in lipid metabolism. ApoE is essential in the formation of chylomicrons, very low-density and high-density lipoproteins (HDL), and is involved in the transport of cholesterol from the peripheral tissues to the liver [1]. The *APOE* gene is located on chromosome 19 and encodes three alleles, ε2, ε3 and ε4, which have been shown to affect the lipid profile and the development of coronary artery disease (CAD) [2, 3]. The distribution of these alleles varies between ethnic groups: Europeans and African-Americans have a high frequency of ε4; Asians have a low frequency of ε2 and ε4 [2].

In the multi-ethnic population of Guadeloupe, a French Caribbean island, about 80% of the population are of African descent, about 8% of Indian descent and about 4% of Caucasian descent. Despite the high prevalence of several cardio-vascular risk factors such as type 2 diabetes (8.1%), hypertension (29.2%) and obesity (22.9%) in the general population [4, 5], data from the regional health observatory indicate a lower mortality rate from ischaemic heart disease (by 50% in men and 40% in women) compared with mainland France [6]. This finding has also been reported in Afro-Caribbean people living in the United Kingdom [7, 8] and may reflect, in part, a more favourable lipid profile in Afro-Caribbean people even in the context of insulin resistance or diabetes [9].

In a study of a sample of the general population of Guadeloupe, Foucan et al. reported a low frequency of hypercholesterolaemia (total cholesterol concentration ≥ 5.2 mmol/L in 38.7% and ≥ 6.2 mmol/L in 11.7% of the sample population) compared with mainland France and other industrialized countries [10]. However, the influence of genetic variants of *APOE* on the metabolic profile and CAD occurrence has never been reported in the Afro-Caribbean population.

The aim of this study was to evaluate the distribution of *APOE* polymorphisms and their association with CAD, lipid parameters and other cardio-metabolic risk factors in Afro-Caribbeans.

## Materials and methods

### Study population

We performed a prospective study that included 754 Afro-Caribbeans (234 CAD patients and 520 non-CAD patients) living on French West Indies islands. The CAD group included patients hospitalized for CAD or those with a documented history of acute coronary syndrome, coronary stenosis, coronary angioplasty or coronary artery by-pass surgery. Non-CAD controls were selected from two units of medicine at the university hospitals of Guadeloupe and Martinique (a neighbouring French island) and a medical health centre in Guadeloupe. Both islands share the same history, have, approximately 420 000 inhabitants, similar socioeconomic status, and ethnic distribution with a majority of Afro-Caribbeans. Controls were deemed free of cardio-vascular disease including myocardial infarction, acute coronary syndrome, peripheral artery disease and stroke. The ethnic origin was determined when the patient defined him/herself and his/her two first-degree relatives as Afro-Caribbean. The study was approved by the regional ethics committee (Sud-Ouest/Outre-Mer III, France). All patients gave their written informed consent to participate in this study.

### Data collection

Data on age, sex, height, weight and use of antihypertensive and/or hypolipidaemic treatment were collected during the enrollment visit. Body mass index (BMI) was calculated as weight divided by height squared (kg/m^2^). Waist circumference (WC) was measured at the midpoint between the lowest rib margin and the top of the iliac crest at minimal respiration with the participant standing. Systolic and diastolic blood pressure was assessed using an automated monitor after the subject had rested for at least 5 min. The recorded values were the average of two measurements in each arm.

### Biochemical analyses

Collection of blood samples was done within a week (generally less than 24h) after the formal enrollment. Blood samples were collected after an overnight fast of > 10 h. The concentrations of glucose, total cholesterol, HDL and triglycerides were measured using an automatic multichannel chemical analyser (Cobas Integra 800, Roche Diagnostic, France). Low-density lipoprotein (LDL) cholesterol concentration was calculated using the Friedewald formula in subjects with triglyceride level < 3.9 mmol/L (340 mg/dL) and the Planella formula based on apolipoprotein B when the triglyceride level was ≥ 3.9 mmol/L.

### Determination of *APOE* isoforms

Genomic DNA was extracted from whole blood cells using a QIAamp^®^ DNA purification kit (Qiagen, France), and DNA content was quantified using NanoVue Plus^™^ (GE Healthcare Life Sciences, France). Genotyping was performed using published TaqMan methods (Applied Biosciences). Genotypes were assigned according to the combinations of the two single-nucleotide polymorphism allelic forms (rs7412 and rs429358) of the *APOE* gene, known as ε2, ε3 and ε4.

### Definition of clinical factors

Obesity was defined as a BMI ≥ 30 kg/m^2^. Dyslipidaemia was defined as a history of blood lipid abnormality and/or receiving lipid-lowering treatment. Hypertension was defined as a systolic blood pressure ≥ 140 mmHg or a diastolic blood pressure ≥ 90 mm Hg, or current use of antihypertensive medication.

### Statistical analyses

The data are presented as mean ± standard deviation for continuous variables and as numbers (percentages) for categorical variables. Student’s *t* test or one-way analysis of variance (ANOVA) were used to compare means and F-test and Levene's test were used for variance equality. T-test and ANOVA assumptions were satisfied for all variables.

The chi-squared test or Fisher’s exact test was used to compare proportions. Triglyceride values were log_10_ transformed to approach a normal distribution before statistical analysis.

Only participants with complete genotype (712 of 754) were included in the study. The proportion of successful *APOE* genotyping was similar in the subjects with CAD (0.95) and the subjects without CAD (0.94).

Subjects were divided into three groups according to their genotype: ε2+ (ε2/ε2, ε2/ε3), ε3 homozygotes (ε3/ε3) and ε4+ (ε3/ε4, ε4/ε4). One-way analysis of variance (ANOVA) and the Bonferroni post hoc test were used to compare the lipid profile and cardio-vascular risk factors between the groups. All tests were two-sided and a p value < 0.05 was considered significant. IBM SPSS Statistics for Windows, Version 19.0 (IBM Corp., Armonk, NY) was used to analyse the data.

## Results

### Characteristics of CAD and non-CAD patients

Table 1 shows the characteristics of the participants according to CAD status. Diabetes, hypertension and dyslipidaemia were significantly more prevalent in the CAD group compared with the control group. Patients with CAD were also older and had a larger WC. The proportion of obese subjects did not differ between the two groups.

**Table 1:** Characteristics of the participants according to CAD status.

	Non-CAD N = 492	CAD N = 220	P
Male (%)	205 (41.7%)	141 (64.1%)	<0.001
Age (years)	51.11(±13.43)	63.51(±10.46)	0.004
Diabetes mellitus (%)	61 (12.4%)	110 (50%)	<0.001
Hypertension (%)	140 (28.5%)	175 (79.5%)	<0.001
Dyslipidemia (%)	78 (15.9%)	112 (50.9%)	<0.001
Smoking	70 (14.3%)	59 (26.8%)	<0.001
Lipid-lowering medication (%)	39 (8%)	166 (77.9%)	<0.001
**Anthropometric**			
Waist circumference	90.4 (±13.8)	98.54 (±11.8)	<0.001
BMI (kg/m^2^)	27.3 (±5.9)	27.4 (± 4.7)	0.831
Obesity (BMI ≥ 30 kg/m2)	143 (29.4%)	50 (22.9%)	0.077
**Genotype (%)**			0.762
ε_2_/ε_2_	5 (1%)	2 (0.9%)	
ε_2_/ε_3_	53 (10.8%)	27 (12.3%)	
ε_2_/ε_4_	19 (3.9%)	5 (2.3%)	
ε_3_/ε_3_	241 (49%)	100 (45.4%)	
ε_3_/ε_4_	150 (30.5%)	76 (34.6%)	
ε_4/_ε_4_	24 (4.9%)	10 (4.5%)	
**Allele frequency**			0.972
ε_2_	8.3	8.2	
ε_3_	69.6	68.9	
ε_4_	22.1	22.9	

Data are presented as means ± SD or number (percentage)

The Fisher’s exact test was used to compare genotype distribution and the chi-squared test to compare allele frequencies and other proportions, among CAD and non-CAD groups.

The proportion of successful *APOE* genotyping was similar in subjects with or without CAD (0.95 and 0.94 respectively). The distribution of the *APOE* polymorphism genotypic frequencies was within the Hardy—Weinberg equilibrium in both groups. In the total sample, the frequencies of the ε2, ε3 and ε4 alleles were 8%, 70% and 22%, respectively. Most subjects were ε3/ε3 homozygotes (n = 341; 48%), 284 subjects (40%) were ε4 carriers (carrying at least one ε4 allele) and 111 (16%) were ε2 carriers (carrying at least one ε2 allele). The *APOE* genotype distribution did not differ significantly between the CAD and the non-CAD groups (Tables 1 and 2). Individuals with the ε2/ε4 genotype (n = 24) were excluded from the subsequent analysis.

**Table 2:** Association between APOE genotype, cardiovascular risk factors, and CAD.

Multivariate logistic regression			
	O R	95% CI	P
**Age**	1.06	(1.04–1.08)	<0.001
**Male gender**	3.48	(2.18–5.56)	<0.001
**Hypertension**	5.19	(3.23–8.36)	<0.001
**Diabetes**	2.16	(1.32–3.53)	0.002
**Hypercholesterolemia**	3.49	(2.22–5.51)	<0.001
**Smoking**	3.03	(1.72–5.34)	<0.001
***APOE*-ε4 carrier**	1.11	(0.71–1.73)	0.648

### Lipid profile and *APOE* polymorphism in non-CAD patients

For non-CAD patients who were not receiving a lipid-lowering treatment (n = 425), the concentrations of total cholesterol, LDL-cholesterol and triglycerides differed significantly between the three genotype groups (Table 3, S1 Fig). *APOE*-ε4 carriers had a higher total cholesterol level compared with ε2 carriers: 5.07 vs 4.59 mmol/L (P = 0.016). LDL levels were significantly different in *APOE*-ε2 carriers, ε3 homozygotes and ε4 carriers: 2.65 vs 3.03 and 3.17 mmol/L, respectively (P = 0.002). *APOE*-ε2 carriers had a higher triglyceride level compared with ε3 homozygotes and *APOE*-ε4 carriers: 1.38 vs 1.08 and 1.07 mmol/L, respectively (P = 0.012 and P = 0.015). The mean HDL-cholesterol level was non-significantly higher in *APOE*-ε4 carriers compared with ε3 homozygotes and ε2 carriers: 3.17 vs 3.03 and 2.65 mmol/L, respectively (P = 0.171). The ratios of total cholesterol/HDL and LDL/HDL did not differ significantly between the three allelic groups.

**Table 3:** Association between lipid levels and *APOE* genotypes in non-CAD subjects.

	All N = 425	ε2+ N = 56	ε3/ε3 N = 215	ε4+ N = 154	P
**Total-C (mmol/L)**	4.90 (±1.08)	4.59 (±1.19)	4.87 (±1.06)	5.07 (±1.05) ^a^	0.015
**LDL-C (mmol/L)**	3.03 (±0.96)	2.65 (±0.88)	3.03 (±0.93) ^b^	3.17 (±0.98) ^c^	0.002
**HDL-C (mmol/L)**	1.42 (±0.63)	1.33 (±0.62)	1.39 (±0.58)	1.49 (±0.70)	0.171
**Total-C/HDL-C**	3.89 (±1.88)	4.12 (±3.63)	3.91 (±1.49)	3.79 (±1.36)	0.520
**LDL-C/HDL-C**	2.46 (±1.33)	2.44 (±2.37)	2.47 (±1.10)	2.44 (±1.08)	0.973
**Triglycerides (mmol/L)**	1.12 (±0.68)	1.38 (±0.88)	1.08 (±0.60) ^d^	1.07 (±0.70) ^e^	0.008

Data are presented as means ± SD and were compared between groups using one-way ANOVA and the Bonferroni post hoc test. Only data from non-CAD patients who were not receiving lipid-lowering medication were considered in the analysis.

^a^ P = 0.016 for ε4+ vs ε2+;

^b^ P = 0.019 for ε3/ε3 vs ε2+;

^c^ P = 0.001 for ε4+ vs ε2+;

^d^ P = 0.012 for ε3/ε3 vs ε2+;

^e^ P = 0.015 for ε4+ vs ε2+

Total-C: total cholesterol; HDL-C: HDL-cholesterol; LDL-C: LDL-cholesterol

### Association between other cardio-metabolic risk factors and *APOE* allele

No significant associations were observed between *APOE* polymorphism and diabetes, hypertension, WC or BMI (Table 4).

**Table 4:** Association between *APOE* genotypes and other cardio-metabolic risk factors.

	Carrier Group			
	ε2+ N = 87	ε3/ε3 N = 341	ε4+ N = 260	P
**Male gender %**	57.5	50.4	42.7	0.033
**Age (SD)**	54.7 (±12.4)	55.1 (±14.1)	55.3 (±14.2)	0.944
**Hypertension %**	36.8	44.0	46.9	0.255
**Diabetes %**	23.0	24.0	25.8	0.831
**BMI (SD)**	27.4 (±5.6)	27.1(± 5.4)	27.6(±5.9)	0.484
**BMI>30%**	24.4	26.8	30.0	0.537
**Waist circumference (SD)**	94.5 (±13.1)	92.3 (±13.7)	92.9 (±14.3)	0.439

BMI: Body mass index

## Discussion

### Allelic distribution of *APOE* in Afro-Caribbeans

The wide variation in the distribution of the *APOE* phenotype and *APOE* alleles between ethnic groups is well established [1]. This study is the first to assess the frequency of *APOE* alleles and to analyse its association with the lipid profile, cardio-vascular risk factors and CAD in Afro-Caribbeans. We found higher frequencies of the ε4 allele (22%) and lower frequencies of the ε3 allele (70%) in the studied sample compared with most other populations described in the literature. Higher frequencies of the non-ε3 allele form and especially the ε4 allele have been reported in African-Americans compared with Asians and Caucasians and in Africans compared with African-Americans [11–14]. Our results indicate that the *APOE* allelic distribution in Afro-Caribbeans is close to that in the African-American population.

### Association of *APOE* polymorphism with the lipid profile

*APOE-*ε4 carriers had the highest total cholesterol concentration whereas *APOE*-ε2 carriers had the lowest LDL and the highest triglyceride concentrations. These findings are consistent with previous studies in different populations [15, 16], some of which suggested that the relationship between the ε2 allele and LDL or total cholesterol concentration might be stronger in people of African descent compared with Caucasians [17, 18].

Most studies and meta-analyses have indicated that ε4 carriers have a lower HDL level compared with ε3 homozygotes and ε2 carriers [16]. In our study, the mean HDL concentration was higher in ε4 carriers compared with other allelic groups, although the difference was not significant. Although the ε2 allele was associated with a lower LDL concentration compared with the ε3 and ε4 alleles, the relative and absolute differences between the three allelic groups were low. As a consequence, the LDL/HDL and total cholesterol/HDL ratios, which reflect the atherogenicity of the lipid profile, were similar in the ε2+, ε3/ε3 and ε4+ participants in this study. This finding is unusual but is consistent with the observation that the effect of *APOE* polymorphism on the blood lipid profile may vary with ethnic, genetic and environmental factors [1, 19–21]. In a study including 207 healthy volunteers, Loktionov et al. reported lower, total cholesterol, LDL cholesterol and total cholesterol/HDL ratio in black South Africans compared to United Kingdom Caucasians with similar *APOE* genotypes [22].

### Association of *APOE* polymorphism with CAD

We found no association between *APOE* gene variants and CAD. Previous epidemiological studies have investigated the relationship between *APOE* polymorphism, plasma concentrations of total cholesterol, LDL and apolipoprotein B, and CAD risk. Most of these studies have reported a significant association of between the *APOE-*ε2/ε3/ε4 genotype and susceptibility to CAD. The *APOE-*ε2 allele seems to be associated with a lipid profile that confers a lower coronary risk and that may be driven by compensatory upregulation of LDL receptors [16]. In contrast to *APOE-*ε2, *APOE-*ε4 is usually associated with high risk of cardio-vascular disease and CAD [2]. This association could be explained, at least partially, by the atherogenic lipid profile reported in ε4 allele carriers. Although we found significant differences in lipid concentrations according to the *APOE* allele, no allelic form appeared to be associated with a particularly adverse profile in our sample. The ε4 allele was associated with a high HDL concentration and low total cholesterol/HDL and LDL/HDL ratios. These variables did not differ according to *APOE* allele. The total cholesterol/HDL and LDL/HDL ratios are strong predictors of the risk of coronary heart disease, even stronger than LDL or HDL levels alone [23–25]. Thus, the association of *APOE* polymorphisms with CAD could be weak or non-existent in the absence of differences in the total cholesterol/HDL and LDL/HDL ratios between allelic groups. This hypothesis is supported by the results of a large prospective cohort study that reported that the association of *APOE* genotype with CAD was no longer significant after adjustment for various cardio-vascular risk factors and especially for the LDL/HDL ratio [26]. The absence of an unfavourable influence of the ApoE genetic variants on the lipid profile combined with the relatively low LDL concentration in Afro-Caribbeans may explain the lack of association between *APOE* polymorphism and CAD.

The low prevalence of smoking in the study sample as well as in our general population is another characteristic that might explain the lack of association between *APOE* polymorphism and CAD. Some studies, have reported a significant interactive effect between *APOE* genotype and smoking on CAD and that the presence of the ε4 allele is associated with an increased risk of CAD exclusively or mainly in smokers [27–30]. This interaction may be attributable to the reduced antioxidant capacity of apoE4 and/or higher levels of LDL and small dense LDL in *APOE*-ε4 carriers, which make them vulnerable to the effects of reactive oxygen species produced by smoking [28, 31]. However, such an interaction between smoking and the *APOE*-ε4 allele has not been observed in other studies or meta-analysis [26, 32].

### Association of *APOE* polymorphism with other cardio-metabolic risk factors

The association between *APOE* gene variants and other cardio-vascular risk factors, such as obesity, diabetes and the metabolic syndrome has also been studied in different ethnic groups. A meta-analysis of 45 studies including 13 940 cases and 16 364 controls by Stoumpos et al. indicated an increased risk of hypertension associated with the ε4 allele compared with the ε2 and ε3 alleles [33]. This association was stronger in populations of Asian descent, with an odds ratio (OR) of 1.49 (95% confidence interval (CI): 1.05–2.13) vs an OR of 1.39 (95% CI: 1.12–1.72) for the overall population (mostly of European descent). However, none of the studies included in this meta-analysis was conducted in an African population or of African descent and few included black subjects.

Although several studies have reported an association between *APOE* polymorphism and obesity, insulin resistance and the metabolic syndrome, the results are inconsistent [34–36]. We found no association between *APOE* polymorphism and any of the traditional cardio-vascular risk factors (except lipid levels) including hypertension, diabetes, smoking, BMI, obesity, waist, age and sex. This lack of association of *APOE* alleles with the cardio-metabolic risk factors measured in our study might reflect the specific effects of these alleles on blood lipid levels and environmental factors such as diet in Afro-Caribbeans. Several studies have indicated that ApoE isoforms may be associated with obesity and its related disorders through direct and indirect effects on lipid metabolism and that obesity and dyslipidaemia play a pivotal role in the development of the metabolic syndrome [35, 37, 38]. The exact role of ApoE isoforms and *APOE* polymorphism in the development of these cardio-metabolic abnormalities remains unclear.

Our study had some limitations. Because of the limited sample size, we cannot rule out that a weak association between *APOE* and CAD may have been undetected because of a lack of statistical power. Moreover, because most of the CAD patients were receiving lipid-lowering treatment at the time of inclusion, the analysis of the lipid profile in relation to the allele distribution focused only on non-CAD subjects.

## Conclusions

This study provides new data about the metabolic and cardio-vascular profile associated with *APOE* polymorphism in Afro-Caribbeans. Unlike numerous studies in other populations, we found no association between *APOE* polymorphism and CAD. Although blood lipid levels were influenced by *APOE* genotype, no allelic form was associated with an atherogenic lipid profile. These findings support the idea that the link between *APOE* polymorphism and CAD varies between different populations and may depend on the effects of *APOE* polymorphism on blood lipid levels.

## Supporting information

S1 Fig Distribution of lipids concentration according to apolipoprotein E genotypes. (DOCX)

## References

[1] Mahley RW, Rall SC Jr., Apolipoprotein E: far more than a lipid transport protein. Annual review of genomics and human genetics. 2000;1:507–37. 10.1146/annurev.genom.1.1.507 . 11701639

[2] Eichner JE, Dunn ST, Perveen G, Thompson DM, Stewart KE, Stroehla BC. Apolipoprotein E polymorphism and cardiovascular disease: a HuGE review. American journal of epidemiology. 2002;155(6):487–95. . 11882522 10.1093/aje/155.6.487

[3] Lahoz C, Schaefer EJ, Cupples LA, Wilson PW, Levy D, Osgood D, et al Apolipoprotein E genotype and cardiovascular disease in the Framingham Heart Study. Atherosclerosis. 2001;154(3):529–37. . 11257253 10.1016/s0021-9150(00)00570-0

[4] Foucan L, Bangou-Bredent J, Ekouevi DK, Deloumeaux J, Roset JE, Kangambega P. Hypertension and combinations of cardiovascular risk factors. An epidemiologic case-control study in an adult population in Guadeloupe (FWI). European journal of epidemiology. 2001;17(12):1089–95. . 12530767 10.1023/a:1021213729434

[5] Inamo J, Daigre JL, Boissin JL, Kangambega P, Larifla L, Chevallier H, et al High blood pressure and obesity: disparities among four French Overseas Territories. Journal of hypertension. 2011;29(8):1494–501. 10.1097/HJH.0b013e328348fd95 . 21720269

[6] Guadeloupe Ordlsd. http://www.orsag.fr/maladies-cardio-vasculaires/63-cardiopathies-ischemiques-guadeloupe.html 2012, accessed 30th april 2015. http://www.orsag.fr/cat/travaux-orsag/pathologies/maladies-cardio-vasculaires.html.

[7] Chaturvedi N. Ethnic differences in cardiovascular disease. Heart. 2003;89(6):681–6. ; 12748237 10.1136/heart.89.6.681 PMC1767706

[8] Wild S, McKeigue P. Cross sectional analysis of mortality by country of birth in England and Wales, 1970–92. Bmj. 1997;314(7082):705–10. ; 9116545 10.1136/bmj.314.7082.705 PMC2126166

[9] Chaturvedi N, McKeigue PM, Marmot MG. Relationship of glucose intolerance to coronary risk in Afro-Caribbeans compared with Europeans. Diabetologia. 1994;37(8):765–72. . 7988778 10.1007/BF00404333

[10] Foucan L, Kangambega P, Ekouevi DK, Rozet J, Bangou-Bredent J. Lipid profile in an adult population in Guadeloupe. Diabetes & metabolism. 2000;26(6):473–80. . 11173718

[11] Sandholzer C, Delport R, Vermaak H, Utermann G. High frequency of the apo epsilon 4 allele in Khoi San from South Africa. Human genetics. 1995;95(1):46–8. . 7814025 10.1007/BF00225073

[12] Willis F, Graff-Radford N, Pinto M, Lawson L, Adamson J, Epstein D, et al Apolipoprotein epsilon4 allele frequency in young Africans of Ugandan descent versus African Americans. Journal of the National Medical Association. 2003;95(1):71–6. ;. 12656452 PMC2594366

[13] Atadzhanov M, Mwaba MH, Mukomena PN, Lakhi S, Mwaba P, Rayaprolu S, et al Frequency of APOE, MTHFR and ACE polymorphisms in the Zambian population. BMC research notes. 2014;7:194 10.1186/1756-0500-7-194 ; 24679048 PMC4230427

[14] Masemola ML, Alberts M, Urdal P. Apolipoprotein E genotypes and their relation to lipid levels in a rural South African population. Scandinavian journal of public health Supplement. 2007;69:60–5. 10.1080/14034950701355635 . 17676504

[15] Hagberg JM, Wilund KR, Ferrell RE. APO E gene and gene-environment effects on plasma lipoprotein-lipid levels. Physiological genomics. 2000;4(2):101–8. . 11120871 10.1152/physiolgenomics.2000.4.2.101

[16] Bennet AM, Di Angelantonio E, Ye Z, Wensley F, Dahlin A, Ahlbom A, et al Association of apolipoprotein E genotypes with lipid levels and coronary risk. Jama. 2007;298(11):1300–11. 10.1001/jama.298.11.1300 . 17878422

[17] Chang MH, Yesupriya A, Ned RM, Mueller PW, Dowling NF. Genetic variants associated with fasting blood lipids in the U.S. population: Third National Health and Nutrition Examination Survey. BMC medical genetics. 2010;11:62 10.1186/1471-2350-11-62 ; 20406466 PMC2876148

[18] Anuurad E, Rubin J, Lu G, Pearson TA, Holleran S, Ramakrishnan R, et al Protective effect of apolipoprotein E2 on coronary artery disease in African Americans is mediated through lipoprotein cholesterol. Journal of lipid research. 2006;47(11):2475–81. 10.1194/jlr.M600288-JLR200 . 16888319

[19] Tan CE, Tai ES, Tan CS, Chia KS, Lee J, Chew SK, et al APOE polymorphism and lipid profile in three ethnic groups in the Singapore population. Atherosclerosis. 2003;170(2):253–60. Epub 2003/11/13. . 14612205 10.1016/s0021-9150(03)00232-6

[20] Ordovas JM, Schaefer EJ. Genes, variation of cholesterol and fat intake and serum lipids. Current opinion in lipidology. 1999;10(1):15–22. . 10095985 10.1097/00041433-199902000-00004

[21] Kolovou GD, Anagnostopoulou KK. Apolipoprotein E polymorphism, age and coronary heart disease. Ageing Research Reviews. 2007;6(2):94–108. 10.1016/j.arr.2006.11.001 17224309

[22] Loktionov A, Vorster H, O'Neill IK, Nell T, Bingham SA, Runswick SA, et al Apolipoprotein E and methylenetetrahydrofolate reductase genetic polymorphisms in relation to other risk factors for cardiovascular disease in UK Caucasians and Black South Africans. Atherosclerosis. 1999;145(1):125–35. . 10428303 10.1016/s0021-9150(99)00022-2

[23] Isles CG, Paterson JR. Identifying patients at risk for coronary heart disease: implications from trials of lipid-lowering drug therapy. QJM: monthly journal of the Association of Physicians. 2000;93(9):567–74. . 10984551 10.1093/qjmed/93.9.567

[24] Kinosian B, Glick H, Preiss L, Puder KL. Cholesterol and coronary heart disease: predicting risks in men by changes in levels and ratios. J Investig Med. 1995;43(5):443–50. . 8528755

[25] Nam B-H, Kannel WB, D’Agostino RB. Search for an Optimal Atherogenic Lipid Risk Profile: From the Framingham Study. The American Journal of Cardiology. 2006;97(3):372–5. 10.1016/j.amjcard.2005.08.055 16442398

[26] Ward H, Mitrou PN, Bowman R, et al Apoe genotype, lipids, and coronary heart disease risk: A prospective population study. Archives of Internal Medicine. 2009;169(15):1424–9. 10.1001/archinternmed.2009.234 19667307

[27] Chaudhary R, Likidlilid A, Peerapatdit T, Tresukosol D, Srisuma S, Ratanamaneechat S, et al Apolipoprotein E gene polymorphism: effects on plasma lipids and risk of type 2 diabetes and coronary artery disease. Cardiovascular diabetology. 2012;11:36 10.1186/1475-2840-11-36 ; 22520940 PMC3372424

[28] Grammer TB, Hoffmann MM, Scharnagl H, Kleber ME, Silbernagel G, Pilz S, et al Smoking, apolipoprotein E genotypes, and mortality (the Ludwigshafen RIsk and Cardiovascular Health study). European heart journal. 2013;34(17):1298–305. 10.1093/eurheartj/eht001 23382465

[29] Gustavsson J, Mehlig K, Leander K, Strandhagen E, Bjorck L, Thelle DS, et al Interaction of apolipoprotein E genotype with smoking and physical inactivity on coronary heart disease risk in men and women. Atherosclerosis. 2012;220(2):486–92. Epub 2011/11/11. 10.1016/j.atherosclerosis.2011.10.011 . 22071360

[30] Talmud PJ, Lewis SJ, Hawe E, Martin S, Acharya J, Marmot MG, et al No APOE effect on coronary heart disease risk in a cohort with low smoking prevalence: the Whitehall II study. Atherosclerosis. 2004;177(1):105–12. 10.1016/j.atherosclerosis.2004.06.008 15488872

[31] Talmud PJ, Stephens JW, Hawe E, Demissie S, Cupples LA, Hurel SJ, et al The Significant Increase in Cardiovascular Disease Risk in APOEɛ4 Carriers is Evident Only in Men Who Smoke: Potential Relationship Between Reduced Antioxidant Status and ApoE4. Annals of human genetics. 2005;69(6):613–22. 10.1111/j.1529-8817.2005.00205.x 16266401

[32] Holmes MV, Frikke-Schmidt R, Melis D, Luben R, Asselbergs FW, Boer JMA, et al A systematic review and meta-analysis of 130,000 individuals shows smoking does not modify the association of APOE genotype on risk of coronary heart disease. Atherosclerosis. 2014;237(1):5–12. 10.1016/j.atherosclerosis.2014.07.038 25173947 PMC4232362

[33] Stoumpos S, Hamodrakas SJ, Anthopoulos PG, Bagos PG. The association between apolipoprotein E gene polymorphisms and essential hypertension: a meta-analysis of 45 studies including 13[thinsp]940 cases and 16[thinsp]364 controls. J Hum Hypertens. 2013;27(4):245–55. 10.1038/jhh.2012.37 22971752

[34] Novotny D, Vaverkova H, Karasek D, Malina P. Genetic variants of apolipoprotein A5 T-1131C and apolipoprotein E common polymorphisms and their relationship to features of metabolic syndrome in adult dyslipidemic patients. Clinical biochemistry. 2014;47(12):1015–21. 10.1016/j.clinbiochem.2014.03.015 . 24709297

[35] Kypreos KE, Karagiannides I, Fotiadou EH, Karavia EA, Brinkmeier MS, Giakoumi SM, et al Mechanisms of obesity and related pathologies: Role of apolipoprotein E in the development of obesity. FEBS Journal. 2009;276(20):5720–8. 10.1111/j.1742-4658.2009.07301.x 19754875

[36] Tao MH, Liu JW, LaMonte MJ, Liu J, Wang L, He Y, et al Different associations of apolipoprotein E polymorphism with metabolic syndrome by sex in an elderly Chinese population. Metabolism. 2011;60(10):1488–96. 10.1016/j.metabol.2011.03.004 21550086

[37] Lee MJ, Chien KL, Chen MF, Stephenson DA, Su TC. Overweight modulates APOE and APOA5 alleles on the risk of severe hypertriglyceridemia. Clinica chimica acta; international journal of clinical chemistry. 2013;416:31–5. 10.1016/j.cca.2012.10.054 . 23178747

[38] Rubin J, Berglund L. Apolipoprotein E and diets: a case of gene-nutrient interaction? Current opinion in lipidology. 2002;13(1):25–32. . 11790960 10.1097/00041433-200202000-00005

