# Supplementary figures and images for: Association of APOE gene polymorphism with lipid profile and coronary artery disease in Afro-Caribbeans
Source: PLoS One. 2017 Jul 20;12(7):e0181620. doi: 10.1371/journal.pone.0181620 (PMC5519172; doi:10.1371/journal.pone.0181620)

S1: Distribution of lipids concentration according to Apolipoprotein E genotypes


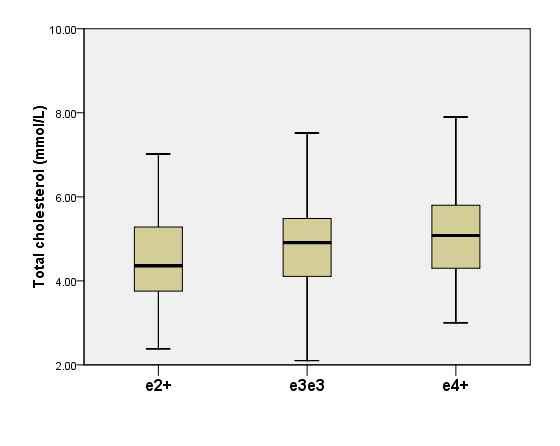

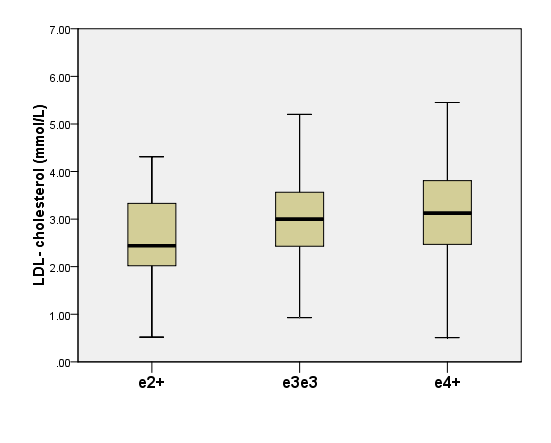

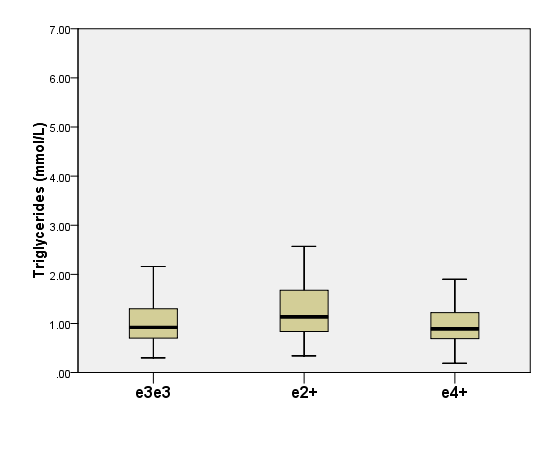

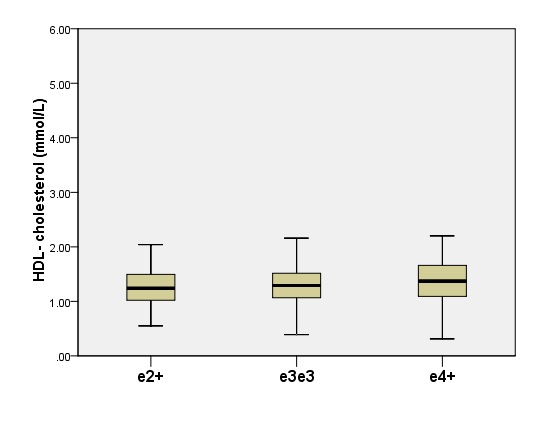

Supplement: S1 Fig — (DOCX) [file pone.0181620.s001.docx]
